# Circadian rhythm disruption and melatonin dysregulation as overlooked drivers of immune imbalance and multiorgan failure in post-COVID syndrome: a call for chronotherapy-based interventions

**DOI:** 10.1097/MS9.0000000000004009

**Published:** 2025-10-07

**Authors:** Ghufran Saeed Rizvi, Rahim Ullah, Maliha Khalid, Muhammad Talha, Aminath Waafira

**Affiliations:** a Department of Medicine, Shifa College of Medicine, Islamabad, Pakistan; b Department of Medicine, Gomal Medical College, D I Khan, Pakistan; c Department of Medicine, Jinnah Sindh Medical University, Karachi, Pakistan; d Department of Medicine, King Edward Medical University, Lahore, Pakistan; e School of Medicine, The Maldives National University, Malé, Maldives

**Keywords:** chronotherapy, circadian rhythm, melatonin, post-COVID syndrome

## Abstract

Post-COVID syndrome (long COVID) is increasingly recognized as a state of chronic inflammation, immune imbalance, and multiorgan dysfunction. Emerging evidence highlights circadian rhythm disruption and melatonin dysregulation as overlooked drivers of persistent symptoms such as fatigue, cognitive impairment, and immune dysregulation. Reduced melatonin impairs cytokine suppression, antioxidant defense, and mitochondrial protection, fueling inflammation and oxidative stress. These disruptions, coupled with autoimmune responses targeting adrenergic and muscarinic receptors, exacerbate systemic pathology. Preliminary data suggest that melatonin supplementation and chronotherapy may restore circadian alignment, rebalance immunity, and mitigate disease progression, although robust large-scale trials remain limited. Integrating circadian science into therapeutic protocols may provide a novel avenue for improving long-term outcomes in post-COVID patients.

*To the Editor,*

Post-COVID-19 syndrome, or long-COVID, affects a significant fraction of individuals recovered from acute SARS-CoV-2 infection and is characterized by persistent symptoms (often coupled with autoimmune manifestations) leading to immune dysregulation and chronic inflammation across multiple organ systems. Recent evidence suggests that circadian rhythm disruption may be a novel predictor of multiorgan failure in these long-haulers, where abnormal sleep–wake cycles and delayed sleep phases can exacerbate fatigue, cognitive impairments, and systemic stress levels. This circadian disruption is thought to result from the virus’s impact on both the central nervous system and inflammatory pathways, which may worsen outcomes in organs like the heart, lung, and kidney by impairing recovery mechanisms. Such circadian misalignment may serve as an early indicator for severe progression and underlines the need to monitor sleep patterns in risk groups^[1]^.

Melatonin, secreted by the pineal gland, regulates circadian rhythms, immunity, and oxidative balance. In post-COVID states, reduced melatonin contributes to persistent inflammation, as suppression of cytokines like TNF-α, IL-6, and IL-8 is impaired. This promotes activation of the NLRP3 inflammasome and JNK pathways, driving tissue damage. Low melatonin also weakens its antioxidant role, worsening oxidative stress, mitochondrial dysfunction, fatigue, and cognitive fog. Its antiviral properties are compromised as well, potentially prolonging viral persistence. Clinical studies suggest melatonin supplementation may restore immune balance, protect mitochondria, and reduce long-term sequelae in long COVID patients^[2]^.

Autoimmunity correlates with circadian rhythm dysregulation in post-COVID syndromes through the targeting of β-adrenergic and muscarinic receptors that exacerbate symptoms such as fatigue and sleep disturbance^[3]^. IL-6 and TNF-α cytokines induce immune dysregulation in the suprachiasmatic nucleus that may result in multiorgan dysfunction^[3]^. Chrono-immunology and melatonin dysregulation are associated with post-COVID multiorgan failure via the hormone’s function to modulate sleep and immune mechanisms^[4]^. The dosage of melatonin therapy depends on phase: 0.5–5 mg for the postinfection, 2–12 mg for the early infection, and 2–12 mg for the pulmonary phase/severe clinical symptoms^[5]^.

Most of the evidence comes from experimental or observational studies, with fewer large-scale clinical trials^[5]^. Melatonin supplementation and chronotherapy have promising results^[5]^, but a standardized dosing schedule, long-term safety profile, and cost-effectiveness analysis are not available. Treatment accessibility, variation in sleep cycle among every individual, and confounding variables such as age, comorbidities, and others also hinder the final interpretation of results. Overall, while encouraging initial results, evidence gaps and methodological shortcomings limit generalizability to its practice and application.

In conclusion, a potential opportunity lies in the restoration of circadian rhythm balance to revolutionize patient care in both COVID survivors and other immune-mediated conditions. Future directions should prioritize standardized clinical protocols and the incorporation of the role of melatonin and related findings into therapy design in post-COVID patients. Multidisciplinary teams of researchers will be crucial in bringing circadian science into everyday healthcare applications. This letter to editor is in compliance with the TITAN guideline^[6]^.

## Data Availability

No data were generated for this manuscript.

## Research registration unique identifying number (UIN)

(1) Name of the registry: N/A. (2) Unique Identifying number or registration ID: N/A. (3) Hyperlink to your specific registration (must be publicly accessible and will be checked): N/A.
